# The Success of Headucate: The Student-Led Mental Health Society

**DOI:** 10.1192/j.eurpsy.2022.292

**Published:** 2022-09-01

**Authors:** V. Selwyn, J. Beezhold, R. Gilmore, R. Howard, I. Bartolome, N. Henderson

**Affiliations:** University of East Anglia, Norwich Medical School, Norwich, United Kingdom

**Keywords:** Schools, Headucate, mental health, Children

## Abstract

**Introduction:**

Headucate: University of East Anglia, a university student-led society, was founded almost 10 years ago by medical students to promote mental health education and raise awareness and funds for mental health causes.

**Objectives:**

Headucate aims to spread mental health awareness and reduce stigma by working with schools, universities, other societies and charities internationally.

**Methods:**

Headucate delivers workshops for children aged 4-18 in primary and secondary schools, community and youth groups and university students. These sessions were delivered in-person pre-COVID and online as interactive webinars since 2020, to spark discussion around mental health, and provide information about the variety of supports available for young people.

**Results:**

In the past decade, Headucate UEA has grown to become one of UEA’s largest student-led groups boasting over 175 members in 2020-2021 from all courses. Within the online world, Headucate’s events have reached worldwide. The initiative has received national recognition, won national student awards and has expanded to set up three further Headucate branches nationwide. Outreach has accelerated and the school workshops reached over 1,000 students in the past year.

**Conclusions:**

Headucate has grown from strength to strength and has plans to continue to develop, with passionate student drivers behind the project. Expansion of the project could include a national mental health university directory, bringing together like-minded mental health advocate students around the country and creating new Headucate branches across the country. To further develop, Headucate could expand outreach to the elderly community as discussed by previous committee.

**Disclosure:**

No significant relationships.

